# Lumbago

**Published:** 1879-09

**Authors:** 


Lumbago.—The treatment of the acute form of lumbago is very simple and very effective. Perhaps the best treatment at first is the application of scarifying cups to the muscle, or muscles affected, to be followed immediately by narcotic fomentations in the shape of a bag of hops soaked in hot water, hot vinegar or alcohol, and applied directly over the scarified parts. There are various stimulating and anodyne liniments which are really excellent in their way—such as turpentine, ammonia, camphor, etc. If opiates are to be employed they should be administered early in the course of the attack. The best form in which to administer opium is in the shape of dover's powder. This may e given in ten grain doses. It is usually very efficient in affording relief to the pain, and at the same time is very likely to produce copious diaphoresis. Where a rapid effect is desired, the opium must be given hypodermically in the shape of morphia.

In most of the cases of lumbago which are encountered in private practice, the patient will be found to object seriously to the use of scarifying cups unless all other remedies are found to be in vain. In fact, you will most of you find in time that the use of this most excellent remedy must be limited to hospital and dispensary cases. Where scarifying cups cannot be employed the best treatment is that by morphia hypodermically, and dover's powder by the mouth. (In the University Hospital, the great pain accompanying lumbago, is at once and very often stopped by the hypodermic injection into the affected muscle of a solution containing one-eightieth of a grain of atropia and one-eighth of a grain of morphia. Great care being always had in the administration of morphia and atropia to nursing women, as belladona is the most powerful anti-galactagogue known, and as too large doses of morphia not infrequently affect the child through its mother's milk.

Another most valuable drug in the treatment of lumbago, is the iodide of potassium which would seem to be clinically proven to have a peculiarly beneficial influence over rheumatism of the lumbar region—more influence over this form of rheumatism, in fact, than over any other. Dr. Graves, of Dublin, is the first one reported to have made use of iodide of potassium in lumbago, and he tried its effects upon his own person. He found that in doses of from five to ten grains, given every three or four hours, its effects were truly wonderful.

This clinical fact—I refer to the peculiar influence of the iodide of potassium upon rheumatism of the lumbar muscles—is very difficult of explanation, but it is undoubtedly true. The iodide has been tried in the treatment of muscular rheumatism of other parts of the body, and its effects, in such cases, have been found to be not by any means so immediately successful.

In the chronic form of lumbago, the condition is one of great obstinacy and is very difficult to treat. Such cases are very apt to persist in disappointing your hopes of cure. The most useful class of remedies here are of course the various forms of counter-irritants, such as blisters, sinapisms, the actual cautery, etc., etc. Thoroughly and conscientiously applied local friction and *massage* may do good in some instances where counter-irritants have signally failed.

Of all remedies, however, for chronic lumbago, I am accustomed to rely mostly upon the influence of tepid water upon the affected parts. The action of water, though slow, is a very permanent one. The water may be applied either in the shape of wet compresses kept in constant contact with the part, or you may use a douche and allow a stream of water to fall steadily upon the rheumatic muscles for some time from a height of from eight to ten feet. This use of water does great good in all forms of muscular rheumatism, no matter where located. After the treatment by douche, or by wet compresses, the parts should be briskly rubbed with a coarse cloth or a skin brush, and then covered with cotton, or wool, or a piece of India-rubber cloth.

I have occasionally derived very advantageous and rapid results from the use of a metallic brush, rubbing the affected part briskly with it. This rubbing acts of course as an electric stimulus, and always give immediate, if not permanent relief, though my experience has been that the use of the electric brush afforded permanent as well as immediate relief.

Very often I advise tying a cloth over the lumbar muscles and ironing them thoroughly, two or three times every day, and then following up the ironing with the application of some stimulating liniment.—*Hospital Gazette and Archives of Clinical Surgery.*

